# Direct and Indirect Effects of UV-B Exposure on Litter Decomposition: A Meta-Analysis

**DOI:** 10.1371/journal.pone.0068858

**Published:** 2013-06-20

**Authors:** Xinzhang Song, Changhui Peng, Hong Jiang, Qiuan Zhu, Weifeng Wang

**Affiliations:** 1 The Nurturing Station for the State Key Laboratory of Subtropical Silviculture and Zhejiang Provincial Key Laboratory of Carbon Cycling and Carbon Sequestration in Forest Ecosystems, Zhejiang A & F University, Lin’an, China; 2 Institute of Environment Sciences, Department of Biology Sciences, University of Quebec at Montreal, Montreal, Canada; 3 Laboratory for Ecological Forecasting and Global Change, College of Forestry, Northwest Agriculture and Forest University, Yangling, China; Portland State University, United States of America

## Abstract

Ultraviolet-B (UV-B) exposure in the course of litter decomposition may have a direct effect on decomposition rates via changing states of photodegradation or decomposer constitution in litter while UV-B exposure during growth periods may alter chemical compositions and physical properties of plants. Consequently, these changes will indirectly affect subsequent litter decomposition processes in soil. Although studies are available on both the positive and negative effects (including no observable effects) of UV-B exposure on litter decomposition, a comprehensive analysis leading to an adequate understanding remains unresolved. Using data from 93 studies across six biomes, this introductory meta-analysis found that elevated UV-B directly increased litter decomposition rates by 7% and indirectly by 12% while attenuated UV-B directly decreased litter decomposition rates by 23% and indirectly increased litter decomposition rates by 7%. However, neither positive nor negative effects were statistically significant. Woody plant litter decomposition seemed more sensitive to UV-B than herbaceous plant litter except under conditions of indirect effects of elevated UV-B. Furthermore, levels of UV-B intensity significantly affected litter decomposition response to UV-B (*P*<0.05). UV-B effects on litter decomposition were to a large degree compounded by climatic factors (e.g., MAP and MAT) (*P*<0.05) and litter chemistry (e.g., lignin content) (*P*<0.01). Results suggest these factors likely have a bearing on masking the important role of UV-B on litter decomposition. No significant differences in UV-B effects on litter decomposition were found between study types (field experiment vs. laboratory incubation), litter forms (leaf vs. needle), and decay duration. Indirect effects of elevated UV-B on litter decomposition significantly increased with decay duration (*P*<0.001). Additionally, relatively small changes in UV-B exposure intensity (30%) had significant direct effects on litter decomposition (*P*<0.05). The intent of this meta-analysis was to improve our understanding of the overall effects of UV-B on litter decomposition.

## Introduction

Ultraviolet-B (UV-B, wavelength between 280–320 nm) has increased by approximately 5% in the last 30 years over northern mid-latitudes and is expected to continue to increase as a result of ozone depletion until the middle of the twenty-first century [1]. Recent research has highlighted numerous ways which UV-B could influence ecological processes, including plant litter decomposition and nutrient release [2,3]. Litter decomposition plays a key role in terrestrial carbon (C) and nitrogen (N) cycling [4,5]. Consequently, UV-B induced changes to litter mass loss could further influence primary production, C storage, and C and nutrient flux between soil and the atmosphere [6].

Previous studies [7–11] have shown that UV-B may affect litter decomposition both directly and indirectly. Direct effects of UV-B exposure refer to how UV-B exposure during litter decomposition may directly alter decomposition rates via induced changes that take place in litter photodegradation or via the abundance, activity, and community composition of decomposers. Indirect effects of UV-B exposure refer to how UV-B exposure during plant growth may alter chemical composition and physical properties of plants and, as a consequence, how these changes will indirectly affect subsequent decomposition processes in soil. Elevated UV-B may directly increase litter decomposition via enhanced lignin photodegradation [7,12,13] or decrease litter decomposition by reducing the abundance and altering the community composition of decomposers [8,14] as well as indirectly accelerating [10,11,15] or slowing [9,12,13] the rate of decomposition via changes in litter chemistry during periods of plant growth. Noteworthy is that some studies have observed no pronounced indirect effects at all [16,17]. The outcome of this is that investigations from assorted experiments are highly variable and difficult to draw general conclusions from with regards to direct and indirect impacts of UV-B exposure on litter decomposition.

Two principal methods used to manipulate UV-B (UV-B supplementation via UV lamps and UV-B reduction via plastic filters) were applied by previous studies investigating UV-B effects on litter decomposition. Although the application of an automated modulated lamp system (for which lamp output is controlled through a feedback cued from ambient solar UV-B) is the most applicable method to use for this type of research, only in a small number of studies chose to apply it [11] due to its high cost and the quality requirements of the lamps themselves. The square wave system (for which lamp output is simply operated by a timer) has been used as an alternative for the bulk of these studies [15,17,18] even though it generally supplies excessive UV-B relative to photosynthetically active radiation (PAR) and, thus, could yield exaggerated effects. Moreover, the UV-B-exclusion method that attenuates the UV-B component in the solar spectrum via plastic filters [2,9,19] could potentially interfere with PAR transmittance and infrared radiation as a result of the range of filters used. Extrapolation of study results must therefore be based on an assumption that plants respond linearly to increasing UV-B levels [20]. When taking this into account, the different approaches used by different experimental methods may result in variations between varying results.

The direction and magnitude of response of litter decomposition to UV-B exposure were also regulated by UV-B level, litter chemistry and shape, decay period length, microbial and faunal communities, and abiotic factors such as precipitation, temperature, and soil structure [2,18]. However, results from these variable responses have not been comprehensively and quantitatively synthesized, which limits our understanding on the role UV-B plays in global biogeochemical cycling. Meta-analysis is a powerful statistical method that compares and integrates results from multiple studies. It has been widely used in evaluating impacts of climate change on forest productivity [21], C sequestration [22], elevated CO_2_ [23,24], N deposition [25–29], and ecological restoration [30] as well as studies associated with invasive species [31,32]. A number of factors that regulate litter decomposition such as N deposition [33] and plant species traits [34] were assessed via the meta-analysis approach. Moreover, meta-analysis has also been applied in the exploration of terrestrial plant [35–37] and aquatic organism [38] response to UV-B exposure. To the knowledge of the authors of this paper, however, no study has been carried out using meta-analysis in the investigation of direct and indirect impacts of UV-B exposure on litter decomposition. In order to characterize the direction and magnitude response of litter decay to UV-B exposure, this study carried out a meta-analysis by synthesizing previous studies carried out throughout the planet’s main biomes (forests, grasslands, deserts, tundra, dwarf shrubs, and fields across North America, South America, Europe, and Asia).

The objectives of this study were to investigate whether the direction and magnitude of direct and indirect effects of UV-B exposure on litter decomposition differ in relation to 1) litter type (woody plant litter vs. herbaceous plant litter), 2) study type (field experiment vs. laboratory incubation), 3) decay period length, 4) UV-B level, and 5) litter form (leaf vs. needle).

## Methods

### 1. Data selection

Data were extracted from peer reviewed publications via a keyword search carried out on “UV-B” or “litter decomposition” or “ultraviolet radiation” from the Web of Science and, specifically, from articles that reported on the effects of UV-B exposure on litter decomposition. For the meta-analysis, studies selected for direct effects were all those related to UV-B exposure during litter decomposition, and for indirect effects were those in which UV-B exposure during plant growth changed the chemical composition of plant foliage and subsequent decomposition in soil was carried out under the controlled conditions (i.e., with no UV-B treatment). Moreover, only data that reported on litter decomposition rates or litter mass loss during UV-B treatments and control experiments were included in this study. The litter decomposition rate and litter mass loss represent the same change in litter decomposition and are therefore often used alternatively in studies related to litter decomposition. To carry out a comprehensive analysis, a total of 26 publications containing 93 data points were selected from 52 articles (tables S1 and S2). An Engauge Digitizer (Free Software Foundation, Inc., Boston, MA, United States of America) was used to extract numerical values from figures in selected articles in which data were graphically presented. Based on methods used to manipulate UV-B levels, the 93 data points were divided into four categories: 1) direct effects of elevated UV-B on litter decomposition, 2) direct effects of attenuated UV-B, 3) indirect effects of elevated UV-B, and 4) indirect effects of attenuated UV-B.

Furthermore, to better understand internal and external factors that regulate the direction and magnitude of litter decomposition response to UV-B, data from each component were subdivided according to litter type (woody plant litter vs. herbaceous plant litter), study type (field experiment vs. laboratory incubation), decay period length, UV-B level, and litter form (leaf vs. needle).

### 2. Meta-analysis

The effect size for each experiment was calculated as the response ratio *r* = *x*_e_/*x*_c_, where *x*_e_ is the mean of the UV-B treatment plots, and *x*_c_ is the mean of the associated control plots.

As is typical in meta-analyses [33,35], most of the articles only reported on mean values of treatment and control plots and not standard deviation or standard error values. To maximize the number of data points in the studies assembled for this analysis, unweighted meta-analysis was applied in much the same way it was applied in previous studies [33,35,39]. The mean effect size for each categorical subdivision was calculated, and a bias-corrected 95% confidence interval (CI) was ascertained by applying the bootstrapping procedure using METAWIN 2.0 [40]. The effect of UV-B exposure on litter decay of a categorical subdivision was considered significant at *P*<0.05 if 95% CI did not overlap 1 [26].

Total heterogeneity among groups (*Q*_t_) was partitioned into within-group heterogeneity (*Q*_w_) and between-group heterogeneity (*Q*_b_). *Q*_b_ for each categorical variable was determined for the response variable. A significance of *Q*_b_ indicated that effect size was different between different categorical subdivisions. Pearson’s correlations between the response ratio of litter decomposition and factors were carried out using SPSS (version 13.0, SPSS Inc., Chicago, Illinois, United States of America) installed on Microsoft Windows.

## Results

**Figure 1:**
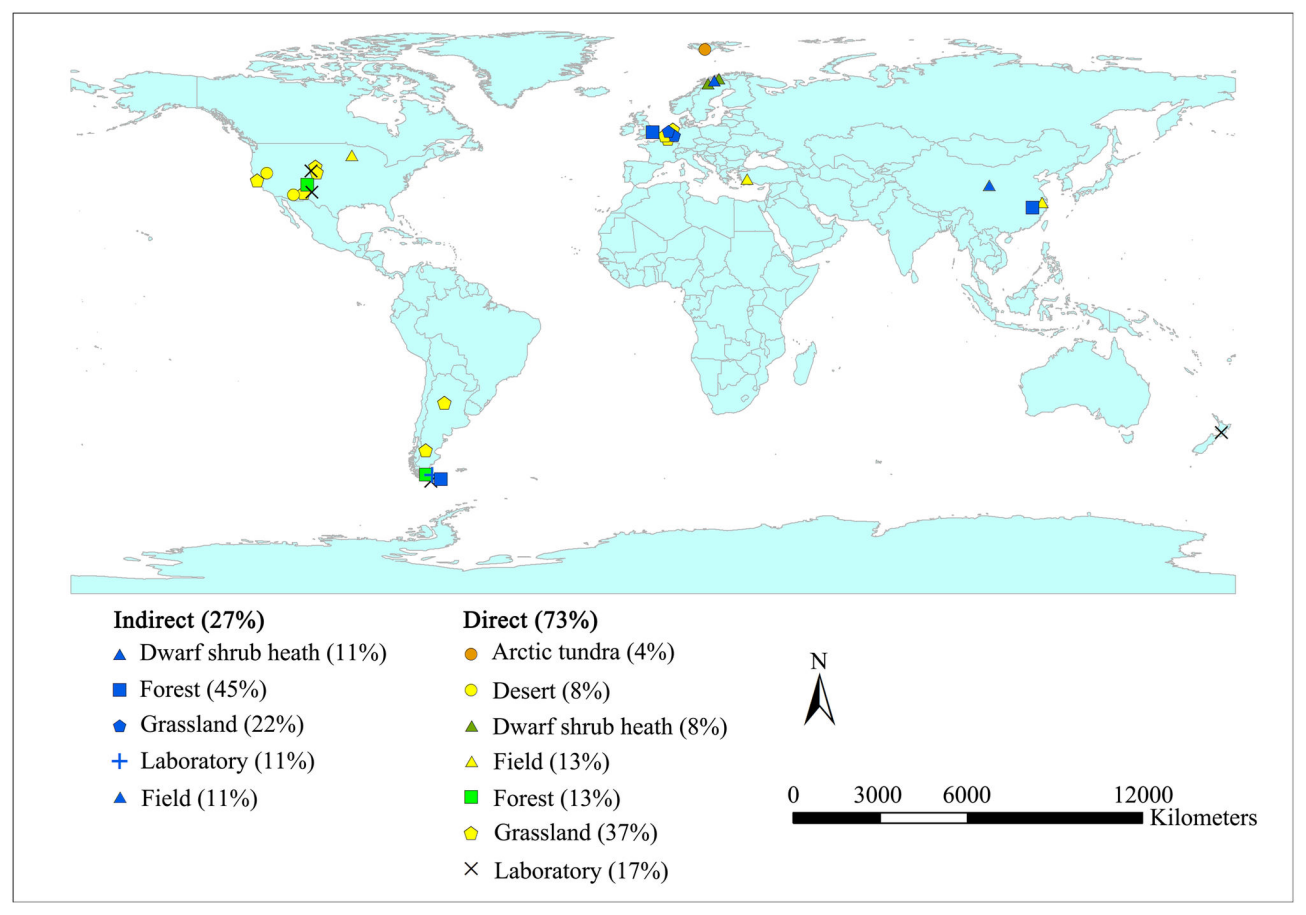
Global distribution of UV-B exposure studies included in meta-analysis. The percental proportion of each major landform type is provided in parenthesis.

Studies on direct effects of elevated and attenuated UV-B on litter decay included 21 and 46 data points, respectively, while studies on indirect effects of elevated and attenuated UV-B on litter decay included 19 and 7 data points, respectively. Experimental sites were situated in forests, grasslands, deserts, tundra, dwarf shrubs, and fields (Figure 1 tables S1 and S2) across North America, South America, Europe, and Asia, primarily within high latitudinal regions. Some laboratory incubations [18,41] were also carried out.

### 1. Direct effects of elevated UV-B

**Figure 2:**
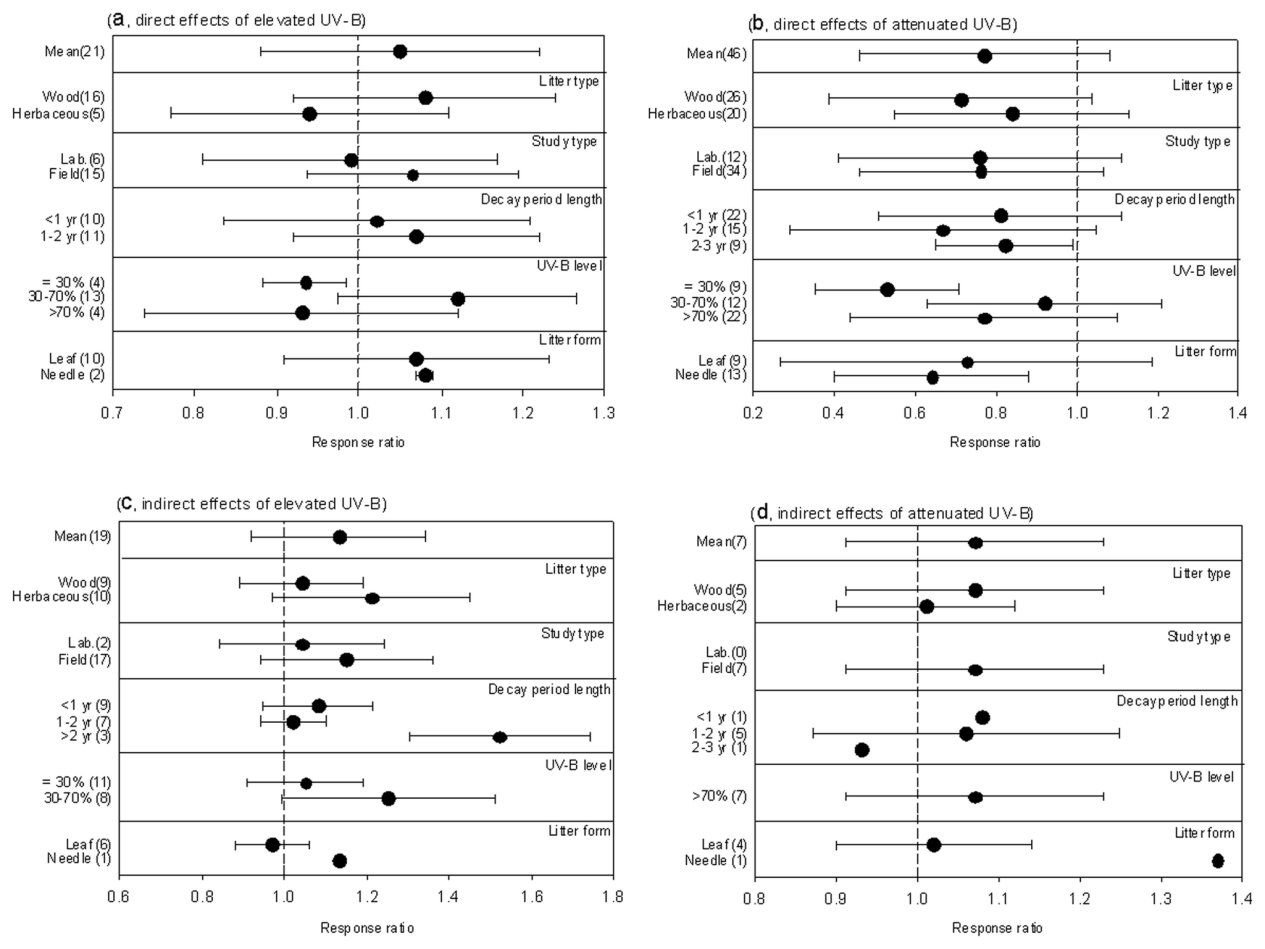
Untransformed response ratios pertaining to UV-B effects on litter decomposition. Direct effects of elevated UV-B (a) and attenuated UV-B (b) as well as indirect effects of elevated UV-B (c) and attenuated UV-B (d) on litter decomposition. Dots with error bars denote the overall mean response ratio with a 95% CI. Wood denotes litter from woody plants; herbaceous denotes litter from herbaceous plants. Lab. denotes the study was carried out in a laboratory; field denotes the study was carried out in the field.

Litter decomposition change ranged from a 32.5% decrease to a 42.9% increase as a direct response to elevated UV-B. On average, overall effects of elevated UV-B on litter decomposition were positive, with a slight decomposition rate increase of 5% (Figure 2a). With an increase of 8%, woody plant litter decomposition response to elevated UV-B was higher than herbaceous plant response, the latter exhibiting a 6% decrease. Results from field experiments showed that decay rates increased by 7% under elevated UV-B treatments while decay rates decreased by 1% under laboratory conditions. All experiments relating to direct effects of elevated UV-B were carried out within a timeframe of less than two years. Direct effects of elevated UV-B did not depend on decay period length, at least during decay processes that transpired within the first two years. A significant decrease of 6% in decomposition rate (*P*<0.05) was determined under conditions of slightly supplemental UV-B (no greater than 30%). Greater supplemental UV-B (greater than 70%) also exhibited a decrease in decomposition rate (7%). However, only an intermediate enhancement in UV-B intensity (between 30% and 70%) greatly accelerated decomposition rates (12%). Both leaf and needle litter decomposition exhibited a positive response to elevated UV-B.

### 2. Direct effects of attenuated UV-B

Attenuated UV-B exposure greatly decreased litter decomposition by an average of 23%, ranging from 69.6% inhibition to 88.7% stimulation (Figure 2b). Litter decomposition rates for both woody and herbaceous plants decreased by 29% and 16%, respectively, under conditions of reduced UV-B exposure. Results from both field and laboratory experiments showed a substantial decline in decay rate. All experiments related to the direct effects of attenuated UV-B were carried out within a timeframe of less than three years (the majority with a timeframe of less than one year). Attenuated UV-B decreased decay rates by 19% during decomposition that transpired over a period of one year and 33% over a period of two years. Moreover, decomposition rates in response to attenuated UV-B decreased by 18% over experimental timeframes lasting between two and three years. A slight reduction in UV-B exposure (no greater than 30%) significantly decreased decay rates (by 47%). In contrast, increased attenuated UV-B exposure (greater than 30%) did not significantly affect litter decomposition, although decay rates decreased by varying degrees. Attenuated UV-B exposure significantly decreased needle litter decay rates (by 36%) but had no significant effect on leaf litter decomposition, from which a 27% decline rate was observed.

### 3. Indirect effects of elevated UV-B

In general, exposure to elevated UV-B during growth periods accelerated subsequent litter decomposition processes (by 13%), ranging from 16.8% inhibition to 77.8% stimulation (Figure 2c). Compared to woody plant litter (exhibiting a 4% increase), herbaceous litter decomposition rates exhibited greater increases (up to 21%). Litter decay rates exhibited a greater positive response to elevated UV-B exposure during growth periods under field conditions compared to laboratory conditions. Decay rates of plant litter exposed to elevated UV-B during growth periods did not significantly increase for decomposition experiments lasting less than two years but significantly increased (by 52%) for decomposition experiments lasting more than two years. Exposure to lower levels of elevated UV-B (≤30% increase) during periods of plant growth slightly accelerated subsequent litter decomposition processes (by 5%) while exposure to higher levels of UV-B greatly accelerated subsequent litter decomposition processes (by 25%). Needle litter decomposition appeared to be more sensitive to elevated UV-B during growth periods compared to leaf litter decomposition.

### 4. Indirect effects of attenuated UV-B

All seven experiments pertaining to indirect effects of attenuated UV-B on litter decomposition were carried out under field conditions with a greater than 70% reduction in levels of UV-B exposure. Study results revealed that exposure to attenuated UV-B during growth periods also accelerated subsequent processes of litter decomposition (by 7%), ranging from 7.7% inhibition to 37.1% stimulation (Figure 2d). Litter decomposition rates for woody and herbaceous plants increased by 7% and 1%, respectively, after exposure to attenuated UV-B during growth periods. Litter decay rates from plants exposed to attenuated UV-B during growth periods increased under decomposition experiments that lasted less than two years but decreased under those that lasted more than two years. Needle litter decomposition appeared to be more sensitive to exposure to attenuated UV-B during growth periods compared to leaf litter.

### 5. Factors controlling litter decomposition response to UV-B exposure

For experiments pertaining to direct effects of both elevated and attenuated UV-B exposure on litter decomposition, only between-group heterogeneity (*Q*_b_) associated with the UV-B change rate proved significant. Other categorical variables such as litter type (woody plant vs. herbaceous plant), study type (field vs. laboratory), decay period length (less than one year, one to two years, and two to three years), and litter form (leaf vs. needle) (Table 1) were not determined significant. For experiments pertaining to indirect effects of elevated UV-B exposure on litter decomposition, only *Q*_b_ associated with decay period length proved significant. The number of data points related to indirect effects of attenuated UV-B exposure was insufficient to analyze *Q*_b_.

**Table 1:** UV-B exposure effects on between-group heterogeneity (*Q*_b_) in relation to the litter decomposition rate.

UV-B treatment	Categorical variable	*Q*_b_	*P*-value	F
Direct effects of elevated UV-B	Litter type	0.074	0.102	2.951
	Experimental conditions	0.013	0.515	0.440
	Decay period length	0.004	0.694	0.162
	**UV-B change rate**	0.133	**0.042**	**5.079**
	Litter form	0.000	0.946	0.005
Direct effects of attenuated UV-B	Litter type	0.179	0.181	1.849
	Experimental conditions	0.004	0.849	0.037
	Decay period length	0.221	0.333	1.130
	**UV-B change rate**	0.774	**0.017**	**4.502**
	Litter form	0.036	0.588	0.303
Indirect effects of elevated UV-B	Litter type	0.139	0.082	3.410
	Experimental conditions	0.087	0.176	1.994
	**Decay period length**	0.564	**0.0001**	**16.93**
	UV-B change rate	0.187	0.040	4.947

Table 2 shows that litter decomposition response to direct effects of elevated UV-B exposure was positively correlated to both mean annual temperature (MAT) and mean annual precipitation (MAP) (*P*<0.01) and that response to direct effects of attenuated UV-B was negatively correlated with both MAP and lignin content (*P*<0.01) and MAT (*P*<0.05). Litter decomposition response to indirect effects of elevated UV-B exposure only showed a positive correlation with decay period length (*P*<0.05). Attenuated UV-B exposure response to indirect effects did not exhibit any significant correlation for any of the five factors: litter type, study type, decay period length, UV-B level, and litter form.

**Table 2:** Pearson’s correlation between the litter decomposition response ratio and extraneous factors involved.

	UV-B treatment	MAT	MAP	Lignin content	Decay period	UV-B change rate
Direct effects	elevated	0.86(8)^**^	0.86(8)^**^	0.58(8)	0.002(15)	0.05(15)
	attenuated	−0.44(23)^*^	−0.53(23)^**^	−0.58(18)^**^	−0.04 (46)	0.21(43)
Indirect effects	elevated	0.66(6)	0.66(6)	−0.37 (5)	0.50(19)^*^	0.49(18)
	attenuated	0.26(7)	0.25(7)	0.28(6)	0.26(7)	0.26(7)

MAT: mean annual temperature; MAP: mean annual precipitation. Values in the brackets denote number of samples.

* p<0.05; **p<0.01.

## Discussion

### 1. Direct effects of UV-B exposure

Results showing how elevated UV-B exposure increased the decay rate (5%) and attenuated UV-B exposure decreased the decay rate (23%) (Figure 2ab) indicated that UV-B may have a general stimulating effect on litter decomposition. Lower sensitivity of litter decomposition to elevated UV-B exposure may indicate that only intermediate enhancement in UV-B intensity (from 30% to 70%) will accelerate decomposition while lower (<30%) or higher (>70%) UV-B exposure intensity restrains decomposition (Figure 2a). Woody plant litter decomposition showed higher sensitivity to both UV-B enhancement and attenuation compared to herbaceous litter (Figure 2ab). This could result from the higher lignin content found in woody litter. Even though lignin only represents a relatively small fraction of total litter composition, it is considered to be a light-absorbing compound that is also resistant to microbial decomposition [42]. Its photodegradation capacity increases the potential for biotic decay of carbohydrates present in litter. A large and growing body of research has shown that lignin photodegradation resulting from UV contributes considerably to the overall decay of surface litter [2,7,43]. Since woody litter typically contains far more lignin than herbaceous litter, it thus exhibits stronger photodegradation tendencies.

*Q*_b_ was not statistically significant for experimental conditions under either elevated or attenuated UV-B treatments (Table 1). This indicates that conclusions from studies carried out under laboratory incubations can be extrapolated to field experiments under certain conditions because litter decomposition investigated in field experiments showed higher sensitivity to UV-B exposure than in laboratory experiments (Figure 2).

Litter decomposition typically undergoes processes that progress from physical to biotic [44]. Leaching of soluble compounds and physical fragmentation play important roles during early stages of decomposition where abiotic factors such as precipitation dominate. UV-B induced photochemical breakdown can facilitate leaching and thus accelerate litter decomposition. Biotic dissociation of the more recalcitrant compounds (such as cellulose, tannins, and lignin) primarily occurs during latter stages for which microbial decomposers and substrate quality are the decisive controlling factors. Although UV-B induced lignin photodegradation accelerates litter decomposition, inhibition on decomposers slows it down. Outcomes will depend on what occurs at the offset of both positive and negative effects. For this study, positive effects were generally yielded. The meta-analysis found that response ratios of litter decay to UV-B exposure during litter decomposition showed no significant differences between decay period lengths (Table 1). This indicated that litter decomposition response to UV-B exposure is insusceptible to duration. In other words, UV-B exposure during litter decay has no significant cumulative effect on litter decomposition.

*Q*_b_ was significant for categories related to both elevated and attenuated UV-B levels (*P*<0.05) (Table 1). This indicated that litter decomposition is sensitive to UV-B exposure. Only intermediate UV-B intensity (from 30% to 70%) accelerated litter decay. Lower (<30%) and higher (>70%) intensities decreased decomposition rates (Figure 2a). A reason for this may be related to the fact that although lower UV-B intensity (<30%) was insufficient for photodegradation to take place, it depressed microbial and faunal communities, thus slowing down rates of decay. Even though photodegradation took place under higher UV-B intensity (>70%), resulting microbial decomposer and faunal activity suppression may counter the positive effects of photodegradation and, thus, exhibit a similar or identical decrease in decay rate. It could be that only through intermediate UV-B intensity (from 30% to 70%) does photodegradation exceed the suppression resulting from microbial decomposer and faunal activity and, as a consequence, stimulate decomposition to the high levels recorded [45]. Furthermore, litter decomposition response to attenuated UV-B was not consistent with UV-B attenuation (Figure 2b). This indicated that litter decomposition response to UV-B exposure level is non-linear. Based on this supposition, the assumption that plants respond linearly to increasing levels of UV-B exposure in studies related to the UV-B-exclusion method [20] should be reconsidered.

For example, one study suggested that ozone recovery is presently taking place, and, because of this, UV-B would unlikely exceed the 30% threshold in the future [46]. Taking this into account, results from experiments that simulate greater than 30% UV-B exposure may not reflect realistic scenarios in terms of evaluating the effects of current or even future UV-B exposure levels on litter decomposition. Moreover, the four experiments that simulated less than a 30% UV-B exposure rate were all carried out in high latitudinal regions (Table S1), indicating that these experiments could be further improved. What these results suggest is that effects of current and future UV-B exposure on litter decomposition remains uncertain.

For litter, *Q*_b_ was not statistically significant under either elevated or attenuated UV-B treatments (Table 1), indicating that no significant difference was detected for either needle and leaf litter decay response to levels of UV-B exposure. The surface area of litter is an important factor impacting photodegradation [45]. With equivalent volume, needle litter typically has a larger surface area than leaf litter and therefore comes into contact with greater levels of UV-B exposure. Needle litter should accordingly be more sensitive to changes in UV-B. The statistical analysis carried out by this study showed that attenuated UV-B exposure significantly decreased needle litter decomposition rates but had no significant effect on leaf litter, testing the above assumption to a certain degree (Figure 2b). However, only two experiments relating to the effects of elevated UV-B on needle litter decomposition were carried out. This would not be considered adequate to confirm or refute this assumption.

### 2. Indirect effects of UV-B exposure

Exposure to elevated UV-B during plant growth accelerates subsequent litter decomposition rate. Moreover, this indirect effect increased with increasing UV-B exposure (Figure 2c) even though *Q*_b_ was not significant for categories of elevated UV-B level (Table 1). Noteworthy was how the response ratio of herbaceous plant litter decomposition exhibited greater sensitivity to UV-B exposure compared to woody plant litter (Figure 2c), which at least (to a certain degree) was likely the result of constituent changes in UV-B absorbing compounds within plant litter growing under supplemental UV-B exposure levels. A plant field study meta-analysis simulating UV-B enhancement [37] pointed out that UV absorbing compounds increased 10% under elevated UV-B exposure levels. A similar meta-analysis [36] also showed an increase of 18.8% and 9.0% in woody and herbaceous plants, respectively. Previous studies have demonstrated that small herbaceous plants generally possess lower contents of UV-B absorbing compounds compared to woody plants and therefore exhibit more rapid response to shifting environmental conditions [36]. Similarly, the present study found that subsequent litter decomposition of herbaceous plants is also more sensitive to growth under conditions of elevated UV-B compared to woody plants.

*Q*_b_ was not significant for study type categories (Table 1), indicating that no significant differences were found for indirect effects of UV-B on litter decomposition between field and laboratory experiments. However, *Q*_b_ was significant for categories related to decay period length (Table 1) and exhibited a trend toward a higher litter decomposition response ratio to UV-B exposure level together with decay duration (Figure 2cd). This highlights the long-term status of indirect effects of UV-B exposure on litter decomposition. As mentioned above, substrate quality is a decisive controlling factor during latter stages of litter decomposition. Exposure to UV-B during plant growth could change the chemical composition of plants [36] and thus the subsequent manner by which litter decomposes in soil. Indirect effects of UV-B exposure would become more obvious during latter stages of litter decomposition and clearly distinguishable from direct effects (Table 1). Even though it was reported that elevated UV-B exposure did not significantly alter biomass, morphology, or physiological variables of woody plants [36], profound and lasting indirect effects of elevated UV-B exposure levels on litter decomposition must be taken into account.

### 3. Factors that work in conjunction with UV-B exposure levels

It is recognized that litter decomposition is a complex process regulated by both biotic and abiotic factors. Less recognized is that direct and indirect effects of UV-B exposure on litter decomposition may interact with other factors such as precipitation [18], litter chemistry [47], and soil [2]. This meta-analysis showed that direct effects of both elevated and attenuated UV-B exposure on litter decay were significantly influenced by both MAT and MAP (*P*<0.05), confirming previous reports that state that MAT and MAP are the critical abiotic factors that control litter decomposition on both regional and global scales [48–50]. Although litter lignin content had a significant effect on the direct response of litter decay to attenuated UV-B exposure (*P*<0.01), no significant effect was found for levels of elevated UV-B exposure (Table 2). This could be partly due to similar negative responses of litter decomposition to different levels of attenuated UV-B (Figure 2ab). After taking into account the combined effects of the biotic and abiotic factors involved, it could be assumed that the important role of UV-B in litter decomposition on a global scale may be masked by factors such as those discussed above, at least to a certain extent. This would be especially true in mesic habitats [51]. Additionally, even if results do not unequivocally establish that indirect effects of UV-B exposure on litter decomposition undergo notable interactions with these factors, the effects were significantly related to decay period length.

### 4. Potential limitations and uncertainties

Although valuable conclusions have been obtained from this estimation on direct and indirect effects of UV-B exposure on litter decomposition, it must be noted that potential limitations and uncertainties could affect results. First, studies relating to UV-B effects on litter decomposition were limited in number, and standard deviation or standard error values of results were rarely provided, which weakens the reliability of the present study. Second, earlier investigations were carried out primarily in grasslands and desert habitats in high latitudinal regions in North America, South America, and Europe under arid and semiarid climatic conditions where UV-B is relatively low owing to latitude effects. Only a few studies were carried out in moist or mesic regions and in Asia and Africa (Figure 1) where UV-B is typically higher (such as in tropical savannas). These data gaps, especially the ones related to indirect effects of UV-B exposure, could potentially cause bias and limit the applicability of conclusions drawn from this meta-analysis. Moreover, baseline differences in incident UV-B exposure levels among latitudes also limit general conclusions. It must also be noted that litterbags, used widely in decomposition experiments, may partially block incoming UV-B and passing through fauna [9,52], thus resulting in a potential underestimation of UV-B effects. It stands to reason that supplementing UV-B could also lead to inadvertent UV-A enhancement [11,18]. At the same time, attenuated UV-B could interfere with the passage of precipitation and PAR by filters placed in plots [20]. Finally, shading effects resulting from lamp arrays and frameworks will also impact observed values [11,53]. All these factors may affect result accuracy and yield a number of uncertainties. To improve our understanding of UV-B effects on litter decomposition, further investigations should be carried out in Asia and Africa, especially in moist and mesic regions, applying more accurate methods by which to simulate the real changes that occur via UV-B exposure.

## Conclusions

An important factor in global climate change, UV-B has been shown to accelerate litter decomposition both directly and indirectly even though the effects are not considered significant. Woody plant litter decomposition appeared to be more sensitive to direct effects of UV-B exposure while herbaceous plants appeared to be more sensitive to indirect effects. The study type (field experiment vs. laboratory incubation), litter form (leaf vs. needle), and decay duration did not significantly influence UV-B effects by and large. To a great extent, litter decomposition response to UV-B was influenced by changes in UV-B levels. Lastly, the interaction of UV-B with key climatic factors (e.g., MAP and MAT) and litter chemistry (e.g., lignin content) could significantly affect litter decomposition across different biomes.

## Supporting Information

Table S1 The study site, longitude and latitude, biome, species, study type, MAT, MAP, litter type, litter form, UV-B treatment, change, and duration of data points relating to direct effects of UV-B exposure on litter decomposition. (DOC)

Table S2 The study site, longitude and latitude, biome, species, study type, MAT, MAP, litter type, litter form, UV-B treatment, change, and duration of data points relating to indirect effects of UV-B exposure on litter decomposition. (DOC)
