# Supplementary material for: Direct and Indirect Effects of UV-B Exposure on Litter Decomposition: A Meta-Analysis
Source: PLoS One. 2013 Jun 20;8(6):e68858. doi: 10.1371/journal.pone.0068858 (PMC3688600; doi:10.1371/journal.pone.0068858)
Supplement: Table S1 — (DOC) [file pone.0068858.s001.doc]

**Table S1: The study site, longitude and latitude, biome, species, study type, MAT, MAP, litter type, litter form, UV-B treatment, UV-B change and duration for observations about the direct effects of UV-B on litter decomposition in the meta-analysis**

| Site | latitude | longtitude | Biome | Species | Study type | MAT (℃) | MAP (mm) | Litter type | Litter form | UV-B treatment | UV-B_BE_ | UV-B change | Duration (month) | Response ratio | Reference |
| --- | --- | --- | --- | --- | --- | --- | --- | --- | --- | --- | --- | --- | --- | --- | --- |
| Amsterdam,  Netherlands | 52° N | 4° E | Dune grassland | *Calamagrostis epigeios and Carex arenaria* | F | - | - | H |  | enhancement | 4.8 | 73% | 5.5 | 1.09 | Verhoef *et al*. (2000) |
| Netherlands | 52.5° N | 4.67°E | Dune grassland | *Calamagrostis epigeios* | F | - | - | H |  | enhancement | 5.0 | 50% | 2 | 1.04 | Rozema *et al*. (1997) |
| Netherlands | 52.5° N | 4.67°E | Dune grassland | *Calamagrostis epigeios* | F | - | - | H |  | enhancement | 5.0 | 50% | 2 | 1.02 | Rozema *et al*. (1997) |
| Guelph  Canada |  |  |  | *Brassica napus* | L | - | - | H |  | enhancement |  | 5.5/ 0 | 2 | 0.67 | Duguay *et al*. (2000) |
| Guelph,  Canada |  |  |  | *Brassica napus* | L | - | - | H |  | enhancement |  | 5.5/ 0 | 2 | 0.89 | Duguay *et al*. (2000) |
| Fort Collins  USA | 40.58° N | 105.15° W | Green house | *Populus tremuloides* | L | 26 | Low | W | broad | enhancement | 7.4 | 51% | 6 | 1.43 | Smith *et al*. (2010) |
| Fort Collins  USA | 40.58° N | 105.15° W | Green house | *Populus tremuloides* | L | 26 | Intermdiate | W | broad | enhancement | 7.4 | 51% | 6 | 1.06 | Smith *et al*. (2010) |
| Fort Collins  USA | 40.58° N | 105.15° W | Green house | *Populus tremuloides* | L | 26 | High | W | broad | enhancement | 7.4 | 51% | 6 | 0.96 | Smith *et al*. (2010) |
| monks wood nature reserve | 52° 24′N | 105.15° W | Forest | *Quercus robur* | F | - | - | W | broad | enhancement |  | 30% | 15 | 0.94 | Newsham *et al*. (1997) |
| Adventdalen, Norway | 78°N | 16° E | Arctic tundra | *Betula pubescens* | F | - | - | W | broad | enhancement |  | 27% | 4 | 1.00 | Moody *et al*. (2001) |
| Abisko, Sweden | 68°N | 18° E | Sub-arctic dwarf shrub heath | *Betula pubescens* | F | -0.8 | 304 | W | broad | enhancement |  | 26% | 14 | 0.91 | Moody *et al*. (2001) |
| Amsterdam, Netherlands | 52°N | 4° E | Dune grassland | *Betula pubescens* | F | - | - | W | broad | enhancement |  | 50% | 14 | 1.02 | Moody *et al*. (2001) |
| Patras, Greece | 38°N | 29° E | Outdoor | *Betula pubescens* | F | - | - | W | broad | enhancement |  | 31% | 14 | 0.94 | Moody *et al*. (2001) |
| Abisko, Sweden | 68.21°N | 18.49° E | Sub-arctic dwarf shrub heath | *Vaccinium uliginosum* | L | - | - | W | broad | enhancement |  | 10.0/ 0 | 2 | 1.05 | Gehrke *et al*. (1995) |
| Abisko, Sweden | 68.21°N | 18.49° E | Sub-arctic dwarf shrub heath | *Vaccinium myrtillu* | F | -0.8 | 304 | W | broad | enhancement | 4.6 | 26% | 12 | 0.89 | Gehrke *et al*. (1995) |
| Zhejiang,  China | 30.27°N | 119.73°E | Forest | *Cinnamonum camphora* | F | 15.6 | 1420 | W | Broad | enhancement |  | 31% | 12 | 1.17 | Song *et al*. (2012a) |
| Zhejiang,  China | 30.27°N | 119.73°E | Forest | *Cyclobalanopsis glauca* | F | 15.6 | 1420 | W | Broad | enhancement |  | 31% | 12 | 1.28 | Song *et al*. (2012a) |
| Zhejiang,  China | 30.27°N | 119.73°E | Forest | *Cunninghamia lanceolata* | F | 15.6 | 1420 | W | Needle | enhancement |  | 31% | 12 | 1.09 | Song *et al*. (2011a) |
| Zhejiang,  China | 30.27°N | 119.73°E | Forest | *Pinus massoniana* | F | 15.6 | 1420 | W | Needle | enhancement |  | 31% | 12 | 1.07 | Song *et al*. (2011a) |
| Zhejiang,  China | 30.27°N | 119.73°E | Forest | *Schima superba* | F | 15.6 | 1420 | W | Broad | enhancement |  | 31% | 12 | 1.24 | unpublished data |
| Zhejiang,  China | 30.27°N | 119.73°E | Forest | *Castanopsis eyeri* | F | 15.6 | 1420 | W | Broad | enhancement |  | 31% | 12 | 1.28 | unpublished data |
| Tierradel Fuego, Argentina | 54.85° S | 68.6° W | Green house | *Hordeum vulgare* | F | 5.5 | 525 | H |  | attenuation |  | 70% | 29 | 1.00 | Pancotto *et al*. (2005) |
| Colorado  USA | 40.82°N | 104.77° W | Grassland | *Bouteloua gracilis* | F | 11 | 340 | H | 5.0 | attenuation |  | 77% | 36 | 0.96 | Brandt *et al*. (2007) |
| Colorado  USA | 40.82°N | 104.77° W | Grassland | *Bouteloua gracilis* | F | 11 | 165 | H | 5.0 | attenuation |  | 77% | 36 | 0.82 | Brandt *et al*. (2007) |
| Cedar Creek,  USA | 45.4° N | 93.2° W | Old field | *Andropogon gerardii* | F | 5.5 | 663 | H |  | attenuation |  | 74% | 24 | 0.95 | Brandt *et al*. (2010) |
| Cedar Creek,  USA | 45.4° N | 93.2° W | Old field | *Bouteloua gracilis* | F | 5.5 | 663 | H |  | attenuation |  | 74% | 24 | 0.86 | Brandt *et al*. (2010) |
| Central Plains,  USA | 40.8° N | 104.8° W | Shortgrass steppe | *Andropogon gerardii* | F | 8.7 | 291 | H |  | attenuation |  | 74% | 24 | 0.67 | Brandt *et al*. (2010) |
| Central Plains,  USA | 40.8° N | 104.8° W | Shortgrass steppe | *Bouteloua gracilis* | F | 8.7 | 291 | H |  | attenuation |  | 74% | 24 | 0.96 | Brandt *et al*. (2010) |
| Sevilleta,  USA | 34.4° N | 106.9° W | Desert grassland | *Andropogon gerardii* | F | 10.5 | 261 | H |  | attenuation |  | 74% | 24 | 0.50 | Brandt *et al*. (2010) |
| Sevilleta,  USA | 34.4° N | 106.9° W | Desert grassland | *Bouteloua gracilis* | F | 10.5 | 261 | H |  | attenuation |  | 74% | 24 | 0.71 | Brandt *et al*. (2010) |
| Argentina | 45.68° S | 70.27° W | Semi-arid steppe | *Mixed grass* | F | - | 152 | H |  | attenuation |  |  | 18 | 0.76 | Austin *et al*. (2006) |
| Cordoba, Argentina | 31.07° S | 64.52°W | Perennial grassland | *Mixed grass* | F | - | - | H |  | attenuation |  | 16% | 4 | 0.75 | Austin *et al*. (2010) |
| California,  USA | 37.67°N | 122.37° W | Annual grasslands | *Lolium multiflorum* | F | 13.1 | 921 | H |  | attenuation |  | 99% | 9 | 0.38 | Henry *et al*. (2008) |
| California,  USA | 37.67°N | 122.37° W | Annual grasslands | *Lolium multiflorum* | F | 13.1 | 921 | H |  | attenuation |  | 99% | 9 | 0.73 | Henry *et al*. (2008) |
| Nevada USA | 39.85°N | 119.85°W | Desert | *Lepidium latifolium* | F | 13.5 | 173 | H |  | attenuation |  | 78% | 12 | 1.04 | Uselman *et al*. (2011) |
| Palmerston North, New Zealand | 40.35°S | 175.62°E | Laboratory | *Lolium perenne* | L | - | - | H |  | attenuation |  | 27% | 2 | 0.84 | Kirschbaum *et al*. (2011) |
| Palmerston North, New Zealand | 40.35°S | 175.62°E | Laboratory | *Lolium perenne* | L | - | - | H |  | attenuation |  | 40% | 2 | 1.50 | Kirschbaum *et al*. (2011) |
| Palmerston North, New Zealand | 40.35°S | 175.62°E | Laboratory | *Lolium perenne* | L | - | - | H |  | attenuation |  | 59% | 2 | 0.53 | Kirschbaum *et al*. (2011) |
| Palmerston North, New Zealand | 40.35°S | 175.62°E | Laboratory | *Lolium perenne* | L | - | - | H |  | attenuation |  | 82% | 2 | 1.09 | Kirschbaum *et al*. (2011) |
| Palmerston North, New Zealand | 40.35°S | 175.62°E | Laboratory | *Lolium perenne* | L | - | - | H |  | attenuation |  | 99% | 2 | 0.36 | Kirschbaum *et al*. (2011) |
| Tierradel Fuego, Argentina | 54.85° S | 68.58° W | Forest | *Gunnera magellanica* | F | 5.6 | 499 | H | broad | attenuation |  | 70% | 4.6 | 1.35 | Pancotto *et al*. (2003) |
| Albuquerque,  USA | 35.126° N | 106.688° W | Riparian forest | *Pinus edulis* | F | 13 | 250 | W | needle | attenuation |  | 80% | 23 | 0.75 | Gallo *et al*. (2009) |
| Albuquerque,  USA | 35.126° N | 106.688° W | Riparian forest | *Pinus edulis* | F | 13 | Add water | W | needle | attenuation |  | 80% | 23 | 0.65 | Gallo *et al*. (2009) |
| Albuquerque  USA | 35.126° N | 106.688° W | Riparian forest | *Juniperus monosperma* | F | 13 | 250 | W | needle | attenuation |  | 80% | 23 | 0.58 | Gallo *et al*. (2009) |
| Albuquerque,  USA | 35.126° N | 106.688° W | Riparian forest | *Juniperus monosperma* | F | 13 | Add water | W | needle | attenuation |  | 80% | 23 | 0.50 | Gallo *et al*. (2009) |
| Albuquerque,  USA | 35.126° N | 106.688° W | Riparian forest | *Populus deltoides* | F | 13 | 250 | W | broad | attenuation |  | 80% | 16 | 0.62 | Gallo *et al*. (2009) |
| Albuquerque,  USA | 35.126° N | 106.688° W | Riparian forest | *Populus deltoides* | F | 13 | Add water | W | broad | attenuation |  | 80% | 16 | 0.41 | Gallo *et al*. (2009) |
| Sevilleta  USA | 34.37°N | 106.53° W | Green house | *Pinus edulis* | L | 21 | - | W | needle | attenuation |  |  | 8 | 1.20 | Gallo *et al*. (2006) |
| Sevilleta,  USA | 34.37°N | 106.53° W | Green house | *Juniperus monosperma* | L | 21 | - | W | needle | attenuation |  |  | 8 | 0.61 | Gallo *et al*. (2006) |
| Sonoran Desert,  USA | 33.5°N | 111.8° W | Desert | *Larrea tridentata* | F | 17.2 | 10.44 | W | broad | attenuation |  | 70% | 4 | 0.78 | Day *et al*. (2007) |
| Sonoran Desert,  USA | 33.5°N | 111.8° W | Desert | *Larrea tridentata* | F | 17.2 | 10.44 | W | Twig | attenuation |  | 70% | 4 | 0.81 | Day *et al*. (2007) |
| Sonoran Desert,  USA | 33.5°N | 111.8° W | Desert | *Larrea tridentata* | F | 17.2 | 10.44 | W | Leaf + Twig | attenuation |  | 70% | 4 | 0.88 | Day *et al*. (2007) |
| Sonoran Desert,  USA | 33.5°N | 111.8° W | Balcony | *Larrea tridentata* | F | 18.9 | 5.11 | W | broad | attenuation |  | 70% | 5 | 0.86 | Day *et al*. (2007) |
| Sonoran Desert,  USA | 33.5°N | 111.8° W | Balcony | *Larrea tridentata* | F | 18.9 | 5.11 | W | Twig | attenuation |  | 70% | 5 | 1.06 | Day *et al*. (2007) |
| Sonoran Desert,  USA | 33.5°N | 111.8° W | Balcony | *Larrea tridentata* | F | 18.9 | 5.11 | W | Leaf + Twig | attenuation |  | 70% | 5 | 0.87 | Day *et al*. (2007) |
| Nevada,  USA | 39.85°N | 119°23′W | Desert | *control Tamarix* | F | 13.5 | 173 | W | Broad | attenuation |  | 78% | 12 | 1.89 | Uselman *et al*. (2011) |
| Zhejiang,  China | 30.27°N | 119.73°E | Forest | *Cinnamonum camphora* | F | 15.6 | 1420 | W | Broad | attenuation |  | 22% | 12 | 0.67 | Song *et al*. (2012b) |
| Zhejiang,  China | 30.27°N | 119.73°E | Forest | *Cyclobalanopsis glauca* | F | 15.6 | 1420 | W | Broad | attenuation |  | 22% | 12 | 0.43 | Song *et al*. (2012b) |
| Zhejiang,  China | 30.27°N | 119.73°E | Forest | *Cunninghamia lanceolata* | F | 15.6 | 1420 | W | Needle | attenuation |  | 22% | 12 | 0.30 | Zhang *et al*. (2011) |
| Zhejiang,  China | 30.27°N | 119.73°E | Forest | *Pinus massoniana* | F | 15.6 | 1420 | W | Needle | attenuation |  | 22% | 12 | 0.52 | Song *et al*. (2011b) |
| Zhejiang,  China | 30.27°N | 119.73°E | Forest | *Schima superba* | F | 15.6 | 1420 | W | Broad | attenuation |  | 22% | 12 | 0.44 | Song *et al*. (2012b) |
| Zhejiang ,  China | 30.27°N | 119.73°E | Forest | *Castanopsis eyeri* | F | 15.6 | 1420 | W | Broad | attenuation |  | 22% | 12 | 0.47 | Song *et al*. (2012b) |
| Palmerston North, New Zealand | 40.35°S | 175.62°E | Laboratory | *Pinus radiata* | L | - | - | W | Needle | attenuation |  | 27% | 2 | 0.35 | Kirschbaum *et al*. (2011) |
| Palmerston North, New Zealand | 40.35°S | 175.62°E | Laboratory | *Pinus radiata* | L | - | - | W | Needle | attenuation |  | 40% | 2 | 0.52 | Kirschbaum *et al*. (2011) |
| Palmerston North, New Zealand | 40.35°S | 175.62°E | Laboratory | *Pinus radiata* | L | - | - | W | Needle | attenuation |  | 59% | 2 | 0.85 | Kirschbaum *et al*. (2011) |
| Palmerston North, New Zealand | 40.35°S | 175.62°E | Laboratory | *Pinus radiata* | L | - | - | W | Needle | attenuation |  | 82% | 2 | 0.89 | Kirschbaum *et al*. (2011) |
| Palmerston North, New Zealand | 40.35°S | 175.62°E | Laboratory | *Pinus radiata* | L | - | - | W | Needle | attenuation |  | 99% | 2 | 0.65 | Kirschbaum *et al*. (2011) |

MAT, Mean Annual Temperature; MAP, Mean Annual Precipitation; F, Field; L, Laboratory; W, Woody plant; H, Herbaceous plant. UV-B_BE_: actual biologically effective UV-B dose in situ (KJ m^-2^ d^-1^)

-, no data was provided in literature.

**References**

Austin AT, Vivanco L (2006) Plant litter decomposition in a semi-arid ecosystem controlled by photodegradation. Nature 442: 555-558.

Austin AT, Ballaré CL (2010) Dual role of lignin in plant litter decomposition in terrestrial ecosystems. Proc Natl Acad Sci U S A 107: 4618-4622.

Brandt LA, King JY, Hobbie SE, Milchunas DG, Sinsabaugh RL (2010) The role of photodegradation in surface litter decomposition across a grassland Ecosystem precipitation gradient. Ecosystems 13: 765-781.

Brandt LA, King JY, Milchunas DG (2007) Effects of ultraviolet radiation on litter decomposition depend on precipitation and litter chemistry in a shortgrass steppe ecosystem. Glob Chang Biol 13: 2193-2205.

Day TA, Zhang ET, Ruhland CT (2007) Exposure to solar UV-B radiation accelerates mass and lignin loss of Larrea tridentata litter in the Sonoran Desert, Plant Ecol 193: 185-194.

Duguay KJ, Klironomos JN (2000) Direct and indirect effects of enhanced UV-B radiation on the decomposing and competitive abilities of saprobic fungi. Appl Soil Ecol 14: 157-164.

Gallo ME, Porras-Alfaro A, Odenbach KJ, Sinsabaugh RL (2009) Photoacceleration of plant litter decomposition in an arid environment. Soil Biol Biochem 41: 1433-1441.

Gallo M, Sinsabaugh RL, Cabaniss SE (2006) The role of ultraviolet radiation in litter decomposition in arid ecosystems. Appl Soil Ecol 34: 82-91.

Gehrke C, Johanson U, Callaghan TV, Chadwick D, Robinson CH (1995) The impact of enhanced ultraviolet-B radiation on litter quality and decomposition processes in Vaccinium leaves from the Subarctic. Oikos, 72, 213-222.

Henry HAL, Brizgys K, Field CB (2008) Litter decomposition in a California annual grassland: Interactions between photodegradation and litter layer thickness. Ecosystems, 11, 545-554.

Kirschbaum MUF, Lambie SM, Zhou H (2011) No UV enhancement of litter decomposition observed on dry samples under controlled laboratory conditions. Soil Biol Biochem 43: 1300-1307.

Moody SA, Paul ND, Bjorn LO *et al*. (2001) The direct effects of UV-B radiation on *Betula pubescens* litter decomposing at four European field sites. Plant Ecol 154: 29-36.

Newsham KK, McLeod AR, Roberts JD, Greenslade PD, Emmett BA (1997) Direct effects of elevated UV-B radiation on the decomposition of Quercus robur leaf litter. Oikos, 79, 592-602.

Pancotto VA, Sala OE, Cabello M *et al*. (2003) Solar UV-B decreases decomposition in herbaceous plant litter in Tierra del Fuego, Argentina: potential role of an altered decomposer community. Glob Chang Biol 9: 1465-1474.

Pancotto VA, Sala OE, Robson TM, Caldwell MM, Scopel L (2005) Direct and indirect effects of solar ultraviolet-B radiation on long-term decomposition. Glob Chang Biol 11: 1982-1989.

Rozema J, Tosserams M, Nelissen HJM, Heerwaarden L, Broekman RA, Flierman N (1997) Stratospheric ozone reduction and ecosystem processes: enhanced UV-B radiation affects chemical quality and decomposition of leaves of the dune grassland species *Calamagrostis epigeios*. Plant Ecol 128: 284-294.

Smith WK, Gao W, Steltzer H, Wallenstein MD, Tree R (2010) Moisture availability influences the effect of ultraviolet-B radiation on leaf litter decomposition. Glob Chang Biol 16: 484 495.

Song X, Jiang H, Zhang H, Peng C, Yu S (2011a) Elevated UV-B radiation did not affect decomposition rates of needles of two coniferous species in subtropical China. Eur J Soil Biol 47: 343-348.

Song X, Zhang H, Jiang H, Yu S, Zhang Z (2011b) Effect of UV-B radiation on the leaf litter decomposition and nutrient release of *Pinus massoniana*. Sheng Tai Xue Bao 31: 2106-2114.

Song X, Zhang H, Chang SX, Jiang H, Peng C, Yu S (2012a) Elevated UV-B radiation increased the decomposition of *Cinnamonum camphora* and *Cyclobalanopsis glauca* leaf litter in subtropical China. J Soils Sediments 12: 307-311.

Song X, Zhang H, Jiang H, Yu S (2012b) Effects of UV-B Radiation on Leaf Litter Decomposition in Humid Subtropical Region in China. Lin Ye Ke Xue 48: 13-17.

Uselman SM, Snyder KA, Blank RR, Jones TJ (2011) UVB exposure does not accelerate rates of litter decomposition in a semi-arid riparian ecosystem. Soil Biol Biochem 43: 1254-1265.

Verhoef HA, Verspagen JMH, Zoomer HR (2000) Direct and indirect effects of ultraviolet-B radiation on soil biota, decomposition and nutrient fluxes in dune grassland soil systems. Biol Fertil Soils 31: 366-371.
