# Supplementary material for: Direct and Indirect Effects of UV-B Exposure on Litter Decomposition: A Meta-Analysis
Source: PLoS One. 2013 Jun 20;8(6):e68858. doi: 10.1371/journal.pone.0068858 (PMC3688600; doi:10.1371/journal.pone.0068858)
Supplement: Table S2 — (DOC) [file pone.0068858.s002.doc]

**Table S2: The study site, longitude and latitude**, biome, species, study type, MAT, MAP, litter type, litter form, UV-B treatment, UV-B change and duration for observations about the indirect effects of UV-B on litter decomposition in the meta-analysis

| Site | latitude | longtitude | Biome | Species | Study type | MAT (℃) | MAP (mm) | Litter type | Litter form | UV-B treatment | UV-B_BE_ | UV-B change | Duration (month) | Response ratio | Reference |
| --- | --- | --- | --- | --- | --- | --- | --- | --- | --- | --- | --- | --- | --- | --- | --- |
| Lanzhou,  China | 36.04°N | 103.51° E | Cropland | *Triticum aestivum* | F | - | - | H | leaf | enhancement | 8.85 | 29% | 3.3 | 1.08 | Yue *et al*. (1998) |
| Lanzhou,  China | 36.04°N | 103.51° E | Cropland | *Triticum aestivum* | F | - | - | H | leaf | enhancement | 8.85 | 48% | 3.3 | 1.16 | Yue *et al*. (1998) |
| Lanzhou,  China | 36.04°N | 103.51° E | Cropland | *Triticum aestivum* | F | - | - | H | leaf | enhancement | 8.85 | 60% | 3.3 | 1.20 | Yue *et al*. (1998) |
| Lanzhou,  China | 36.04°N | 103.51° E | Cropland | *Triticum aestivum* | F | - | - | H | stem | enhancement | 8.85 | 29% | 3.3 | 1.06 | Yue *et al*. (1998) |
| Lanzhou,  China | 36.04°N | 103.51° E | Cropland | *Triticum aestivum* | F | - | - | H | stem | enhancement | 8.85 | 48% | 3.3 | 1.21 | Yue *et al*. (1998) |
| Lanzhou,  China | 36.04°N | 103.51° E | Cropland | *Triticum aestivum* | F | - | - | H | stem | enhancement | 8.85 | 60% | 3.3 | 1.21 | Yue *et al*. (1998) |
| Heemskerk,  Netherlands | 52.5° N | 4.67° E | Dune grassland | *Carex arenaria* | F | - | - | H |  | enhancement | 5.0 | 50% | 24 | 1.43 | Hoorens *et al*. (2004) |
| Heemskerk,  Netherlands | 52.5° N | 4.67° E | Dune grassland | *Calamagrostis epigejos* | F | - | - | H |  | enhancement | 5.0 | 50% | 24 | 1.78 | Hoorens *et al*. (2004) |
| Netherlands | 52.5° N | 4.67° E | Dune grassland | *Calamagrostis epigeios* | F | - | - | H |  | enhancement | 5.0 | 50% | 2 | 0.96 | Rozema *et al*. (1997) |
| Guelph  Canada | - | - | - | *Brassica napus* | L | - | - | H |  | enhancement |  | 5.5/ 0 | 2 | 1.04 | Duguay *et al*. (2000) |
| monks wood nature reserve | 52.4° N | 0.23° W | Forest | *Quercus robur* | F | - | - | W |  | enhancement |  | 30% | 49 | 1.37 | Newsham *et al*. (2001) |
| monks wood nature reserve | 52.4° N | 0.23° W | Forest | *Quercus robur* | F | - | - | W |  | enhancement |  | 30% | 16 | 1.07 | Newsham *et al*. (1999) |
| Abisko, Sweden | 68.21°N | 18.49° E | Sub-arctic dwarf shrub heath | *Vaccinium myrtillu* | F | -0.8 | 304 | W | broad | enhancement | 4.6 | 26% | 12 | 0.91 | Gehrke *et al*. (1995) |
| Abisko, Sweden | 68.21°N | 18.49° E | Sub-arctic dwarf shrub heath | *Vaccinium uliginosum* | L | - | - | W | broad | enhancement | 4.6 | 26% | 2 | 0.83 | Gehrke *et al*. (1995) |
| Zhejiang, China | 30.23°N | 119.7°E | Forest | *Cunninghamia lanceolata* | F | 15.6 | 1420 | W | Needle | enhancement |  | 10% | 22 | 1.13 | Song *et al*. (2013) |
| Zhejiang, China | 30.23°N | 119.7°E | Forest | *Cinnamonum camphora* | F | 15.6 | 1420 | W | broad | enhancement |  | 10% | 22 | 1.06 | Song *et al*. (2013) |
| Zhejiang, China | 30.23°N | 119.7°E | Forest | *Schima superba* | F | 15.6 | 1420 | W | broad | enhancement |  | 10% | 22 | 1.06 | Song *et al*. (2013) |
| Zhejiang, China | 30.23°N | 119.7°E | Forest | *Cyclobalanopsis glauca* | F | 15.6 | 1420 | W | broad | enhancement |  | 10% | 22 | 1.06 | Song *et al*. (2013) |
| Zhejiang, China | 30.23°N | 119.7°E | Forest | *Elaeocarpus sylvestris* | F | 15.6 | 1420 | W | broad | enhancement |  | 10% | 22 | 1.02 | Song *et al*. (2013) |
| Tierradel Fuego, Argentina | 54.85° S | 68.6° W | Green house | *Hordeum vulgare* | F | 5.5 | 525 | H |  | attenuation |  | 78% | 29 | 0.93 | Pancotto *et al*. (2005) |
| Tierradel Fuego, Argentina | 54.85° S | 68.58° W | Forest | *Gunnera magellanica* | F | 5.6 | 499 | H | broad | attenuation |  | 78% | 4.6 | 1.08 | Pancotto *et al*. (2003) |
| Zhejiang, China | 30.23°N | 119.7°E | Forest | *Cunninghamia lanceolata* | F | 15.6 | 1420 | W | Needle | attenuation |  | 80% | 22 | 1.37 | unpublished data |
| Zhejiang, China | 30.23°N | 119.7°E | Forest | *Cinnamonum camphora* | F | 15.6 | 1420 | W | broad | attenuation |  | 80% | 22 | 1.19 | unpublished data |
| Zhejiang, China | 30.23°N | 119.7°E | Forest | *Schima superba* | F | 15.6 | 1420 | W | broad | attenuation |  | 80% | 22 | 0.99 | unpublished data |
| Zhejiang, China | 30.23°N | 119.7°E | Forest | *Cyclobalanopsis glauca* | F | 15.6 | 1420 | W | broad | attenuation |  | 80% | 22 | 0.99 | unpublished data |
| Zhejiang, China | 30.23°N | 119.7°E | Forest | *Elaeocarpus sylvestris* | F | 15.6 | 1420 | W | broad | attenuation |  | 80% | 22 | 0.92 | unpublished data |

MAT, Mean Annual Temperature; MAP, Mean Annual Precipitation; F, Field; L, Laboratory; W, Woody plant; H, Herbaceous plant. UV-B_BE_: actual biologically effective UV-B dose in situ (KJ m^-2^ d^-1^)

-, no data was provided in literature.

**References**

Duguay KJ, Klironomos JN (2000) Direct and indirect effects of enhanced UV-B radiation on the decomposing and competitive abilities of saprobic fungi. Appl Soil Ecol 14: 157-164.

Gehrke C, Johanson U, Callaghan TV, Chadwick D, Robinson CH (1995) The impact of enhanced ultraviolet-B radiation on litter quality and decomposition processes in Vaccinium leaves from the Subarctic. Oikos, 72, 213-222.

Hoorens B, Aerts R, Stroetenga M (2004) Elevated UV-B radiation has no effect on litter quality and decomposition of two dune grassland species: evidence from a long-term field experiment. Glob Chang Biol 10: 200-208.

Newsham KK, Anderson JM, Sparks TH, Splatt P, Woods C, Mcleod AR (2001) UV-B effect on *Quercus robur* leaf litter decomposition persists over four years. Glob Chang Biol 7: 479-483.

Newsham KK, Greenslade PD, Kennedy VH, Mcleod AR (1999) Elevated UV-B radiation incident on *Quercus robur* leaf canopies enhances decomposition of resulting leaf litter in soil. Glob Chang Biol 5: 403-409.

Pancotto VA, Sala OE, Cabello M *et al*. (2003) Solar UV-B decreases decomposition in herbaceous plant litter in Tierra del Fuego, Argentina: potential role of an altered decomposer community. Glob Chang Biol 9: 1465-1474.

Pancotto VA, Sala OE, Robson TM, Caldwell MM, Scopel L (2005) Direct and indirect effects of solar ultraviolet-B radiation on long-term decomposition. Glob Chang Biol 11: 1982-1989.

Rozema J, Tosserams M, Nelissen HJM, Heerwaarden L, Broekman RA, Flierman N (1997) Stratospheric ozone reduction and ecosystem processes: enhanced UV-B radiation affects chemical quality and decomposition of leaves of the dune grassland species *Calamagrostis epigeios*. Plant Ecol 128: 284-294.

Song X, Zhang H, Jiang H, Donaldson LA, Wang H (2013) Influence of elevated UV-B radiation on leaf litter chemistry and subsequent decomposition in humid subtropical China. J Soils Sediments 13: 846-853.

Yue M, Li Y, Wang X (1998) Effects of enhanced ultraviolet-B radiation on plant nutrients and decomposition of spring wheat under field conditions. Environ Exp Bot 40: 187-196.
